# Supplementary material for: NRG1 is a critical regulator of differentiation in TP63-driven squamous cell carcinoma
Source: eLife. 2019 May 30;8:e46551. doi: 10.7554/eLife.46551 (PMC6606022; doi:10.7554/eLife.46551)
Supplement: Figure 4—source data 1. [file elife-46551-fig4-data1.docx]

| **Panel of human airway basal cell gene signature in Figure 4E (as published by (Hackett et al., 2011)** | | | |
| --- | --- | --- | --- |
| LEPREL1 | SERPINE1 | PLAU | IL1RL1 |
| PHLDA1 | STC2 | TRIB3 | SLC16A1 |
| PSAT1 | ALDH1L2 | KLK7 | LIPG |
| BNC1 | ITGA6 | CAY1 | COL17A1 |
| IL13RA2 | NRG1 | SERPINE2 | THBS1 |
| ADAMTS1 | SLC7A5 | FSCN1 | EREG |
| TUBB6 | CALD1 | EHD2 | CAMK2N1 |
| IL1RN | KRT6A | SPRR1A | SCEL |
| SPRR3 | SPRR1B | KRT16 | DSG3 |
| KRT6B | GJB6 | AREG | GJB2 |
| CRCT1 | UPP1 | RGS20 | DKK1 |

**Figure 4-source data 1**
